# GDNF stimulates the proliferation of cultured mouse immature Sertoli cells via its receptor subunit NCAM and ERK1/2 signaling pathway

**DOI:** 10.1186/1471-2121-11-78

**Published:** 2010-10-18

**Authors:** Yongguang Yang, Chunsheng Han

**Affiliations:** 1 State Key Laboratory of Reproductive Biology, Institute of Zoology, Chinese Academy of Sciences, Beijing 100080, China; 2 Graduate University of the Chinese Academy of Sciences, Chinese Academy of Sciences, Beijing, 100101, China

## Abstract

**Background:**

The proliferation and final density of Sertoli cells in the testis are regulated by hormones and local factors. Glial cell line-derived neurotrophic factor (GDNF), a distantly related member of the transforming growth factor-β superfamily, and its receptor subunits GDNF family receptor alpha 1 (GFRα1), RET tyrosine kinase, and neural cell adhesion molecule (NCAM) have been reported to be expressed in the testis and involved in the regulation of proliferation of immature Sertoli cells (ISCs). However, the expression patterns of these receptor subunits and the downstream signaling pathways have not been addressed in ISCs.

**Results:**

In the present study, we have reported that the proliferation of cultured ISCs was significantly enhanced by GDNF. The receptor subunits GFRα1 and NCAM but not RET were expressed in ISCs, and the stimulatory effect of GDNF on the proliferation of ISCs was significantly reduced by anti-NCAM antibody blocking or siRNA that specifically targets NCAM mRNA. Additionally, the ERK1/2 inhibitor, PD98059, completely abolished the mitogenic effect of GDNF on ISCs.

**Conclusions:**

GDNF stimulates the proliferation of ISCs via its receptor subunit NCAM and the consequent activation of the ERK1/2 signaling pathway.

## Background

Sertoli cells secrete growth factors to regulate the proliferation and differentiation of germ cells and themselves [1]. One such factor is glial cell line-derived neurotrophic factor (GDNF), a distantly related member of the transforming growth factor-β (TGF-β) superfamily [2-5]. GDNF was first identified by its ability to support embryonic midbrain dopaminergic neurons *in vitro *[6]. One type of GDNF receptor complex is composed of a ligand-binding subunit, GFRα1, which is a glycosylphosphatidyl-inositol (GPI)-linked protein that may also be secreted, and a signal transducing subunit RET, a receptor tyrosine kinase [7,8]. GDNF-null mice have defects in their nervous system, lack ureters and kidneys, and die 1-1.5 days after birth although their gonads seem normal [9-11]. GFRα1- and RET-null mice exhibit similar phenotypes as GDNF-null mice and die during the first postnatal day [12,13]. Another GDNF receptor complex is composed of GFRα1 and the p140 isoform of neural cell adhesion molecule (p140 NCAM)[14]. Neural cell adhesion molecule (NCAM)-null mice are healthy and fertile although defects have been noticed in their nervous system [15]. The early death of GDNF-, GFRα1- and RET-null mice after birth prevents further investigation on the potential roles that GDNF may have during spermatogenesis.

The role of GDNF in spermatogenesis is demonstrated more clearly by GDNF^+/- ^mice and by mice with GDNF specifically over-expressed in the testis [5]. Although most GDNF^+/- ^mice survive to adulthood and are fertile, spermatogenesis is disturbed in half of the seminiferous tubules because of spermatogonia reduction or depletion. Testicular morphology of mice over-expressing GDNF is normal at birth. However, large type A spermatogonial clusters start to form 2-3 weeks later, resulting in germ cell apoptosis after puberty and non-metastatic tumors at one year of age. The proliferation and function of the Sertoli cells in both types of mice seem to be unchanged. However, whether the trophic effect of GDNF on spermatogenesis is also mediated by its action on Sertoli cells has not been addressed.

It was reported that GDNF stimulated the proliferation of post-natal day 6 rat Sertoli cells in cultured testicular fragments in the presence of follicular stimulating hormone (FSH) [2]. Other reports have indicated that GDNF stimulated the mitosis of Sertoli cells isolated from developing mouse gonads [3] or neonatal mouse testis [16]. In mice, the mRNAs of GDNF, GFRα1 and RET have been detected in urogenital ridges and testis before and after birth by *in situ *hybridization assays [17,18], and a decrease in their expression was observed after the first post-natal week [5]. Consistently, in rats, GDNF mRNA expression increased until post-natal day 7, and then declined during the second and third post-natal weeks, and was lowest in adult testis [19]. The expression of NCAM was detected in fetal or immature Sertoli cells and was downregulated in the rat testis during the maturation of Sertoli cells [20,21]. However, the question about the expression of GFRα1, RET and NCAM in Sertoli cells has not been conclusively addressed. In the present study, we demonstrated that GDNF stimulated the proliferation of cultured ISCs from pup mice, and this effect was mediated by the NCAM receptor subunit and the downstream ERK1/2 signaling pathway.

## Results

### GDNF stimulates the proliferation of mouse ISCs

**Figure 1:**
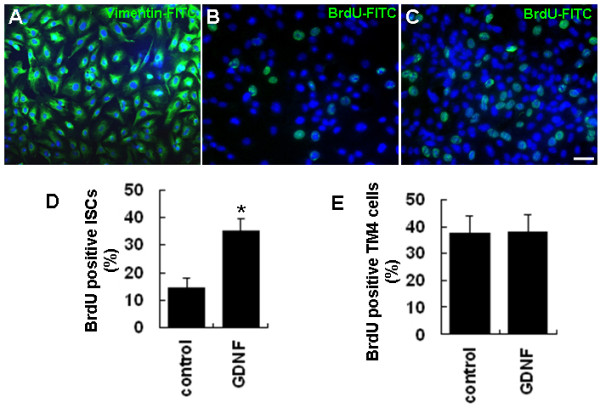
**GDNF enhances the proliferation of cultured ISCs**. (A) The identity and purity of cultured ISCs was confirmed by immunostaining with an antibody against Sertoli cell-specific vimentin protein. (B-C) BrdU-positive ISCs in control (B) and GDNF treated (C) groups. (D) Quantitative analysis of ISC proliferation as indicated by the percentage of BrdU-positive cells in control and GDNF treated groups. (E) Quantitative analysis of TM4 cell proliferation as indicated by the percentages of BrdU-positive cells in GDNF treated and control groups. Statistically significant differences (*p *< 0.05) among groups are indicated by an asterisk. At least three separate experiments were carried out using the ISC and TM4 cells, with 150-200 cells counted in each experiment. Scale bars indicate 10 μm.

Highly purified ISC cultures from 4-5-day-old mice were acquired through several passages of testicular cells, which were maintained in serum-free DMEM/F12 medium. The Sertoli cell-specific protein vimentin [22-25] was detected by immunostaining to evaluate the purity of the culture. After immunocytochemical staining, the numbers of vimentin-positive cells and DAPI stained nuclei were counted. As shown in Figure 1A, more than 95% (data not shown) of the cells were vimentin-positive Sertoli cells.

To test whether GDNF affects the proliferation of ISCs, GDNF (20 ng/ml) was added into the culture medium, and the proliferation of ISCs was evaluated by BrdU incorporation. The results demonstrated that GDNF could significantly stimulate the proliferation of the cultured ISCs. While about 14% of ISCs were BrdU-positive in the control culture, the percentage of BrdU-positive cells increased to 34% when cells were cultured in medium containing 20 ng/ml GDNF for 24 h (Figures 1B,C and 1D). In contrast, there was no significant difference in the percentages of the BrdU-positive cells between the GDNF treated and control groups of the TM4 cell line (Figure 1E), an immortalized mouse Sertoli cell line whose responsiveness to FSH stimulation was reduced [26].

### Expression of GDNF receptor subunits in ISCs and TM4 cells

**Figure 2:**
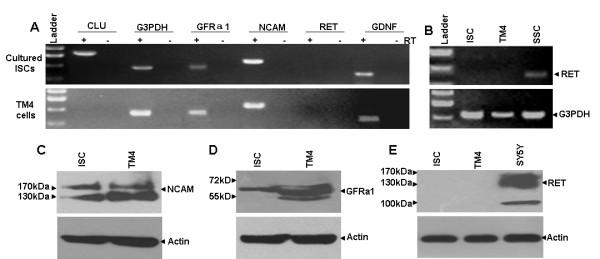
**Expression of GDNF and its receptor subunits in cultured ISCs and TM4 cells**. (A) RT-PCR results exhibited the expression of GDNF, GFRα1 and NCAM but not RET mRNAs in cultured ISCs and TM4 cells. G3PDH and the Sertoli cell-specific gene *CLU *were used as positive controls. (B) RET expression in SSCs but not cultured ISCs or TM4 cells was detected by RT-PCR. (C-E) Western blotting demonstrated the expression of NCAM (C) and GFRα1 (D) but not RET (E) proteins in cultured ISCs and TM4 cells. The SY5Y cell line was used as the positive control for the RET protein. The β-actin protein was used as a positive control for each sample.

The mRNA and protein expression of GDNF receptor subunits GFRα1, RET, and NCAM were investigated by RT-PCR and Western blotting assays, respectively. As shown in Figure 2A, transcripts for GFRα1 and NCAM but not RET were detected in either ISCs or TM4 cells. Transcripts of the Sertoli cell marker gene clusterin (Clu) [27] was only detected in ISCs but not in TM4 cells. To show that the absence of the RET transcript in ISCs and TM4 cells was not a technical artifact, we detected its presence in the cultured spermatogonial stem cells (SSCs) [28] (Figure 2B). Western blotting results showed that protein expression of these three receptor subunits followed the same pattern as their transcripts (Figures 2C,D and 2E). Again, the validity of the RET antibody was demonstrated by its recognition of the antigen in the SY5Y neural cell line [29] as shown in Figure 2E.

### NCAM mediates the proliferation stimulating effect of GDNF on ISCs

**Figure 3:**
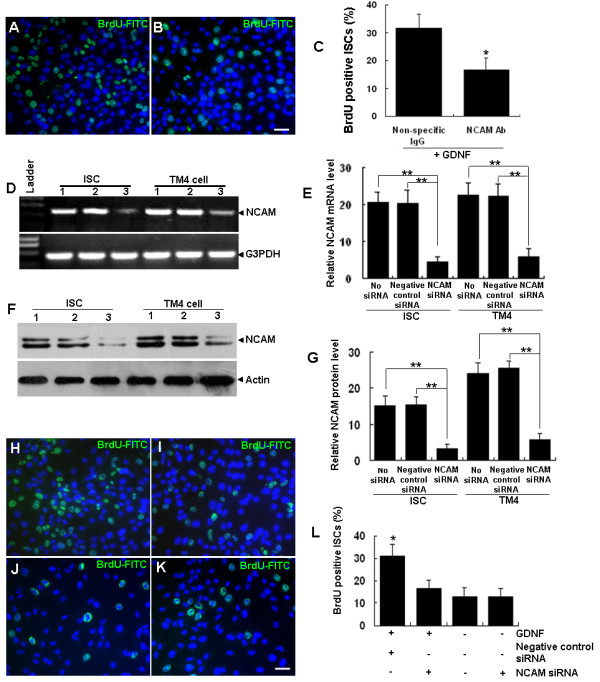
**The proliferation stimulating effect of GDNF on ISCs is mediated by NCAM**. (A-C) BrdU-positive ISCs in response to GDNF stimulation with cells pre-treated with non-specific IgG (A) and NCAM polyclonal antibody (B) and the quantitative comparison (C). (D-G) NCAM mRNA (D, E) and protein (F, G) levels in ISCs and TM4 cells were significantly reduced by NCAM-specific siRNA but not by the negative control siRNA when compared with control groups (1, normal culture control; 2, negative control siRNA; 3, NCAM siRNA). G3PDH and β-actin served as the loading controls for RNA and protein respectively. Expression values of NCAM mRNA (E) or protein (G) were normalized against G3PDH or β-actin signals, respectively. (H-L) BrdU-positive ISCs with negative control siRNA (H) and NCAM siRNA groups (I) stimulated with GDNF and in normal culture controls (J) and the NCAM siRNA group (K) not treated with GDNF, as well as the quantitative comparison among all groups (L). The data is presented as means ± SD from three independent experiments. Statistically significant differences (*p *< 0.05) among groups are indicated by * or ** (*p *< 0.01). Scale bar indicates 10 μm.

Cultured ISCs were pre-treated with a polyclonal human NCAM antibody that was raised against the N-terminal 300 amino acids, and then treated with GDNF and pulse-labeled with BrdU. After immunocytochemical staining, the numbers of BrdU-positive cells and DAPI stained nuclei were counted. As shown in Figures 3A-C, the percentage of the BrdU positive cells in the NCAM antibody-treated group was significantly lower than that in the non-specific IgG treated group. Next, we knocked down the expression of NCAM by siRNAs that specifically targeted the NCAM mRNA, and tested whether the proliferation stimulation effect of GDNF on ISCs could also be abolished. As shown by Figures 3D-G, NCAM mRNA and protein expression in both ISCs and TM4 cells were significantly reduced in the NCAM siRNA transfected group compared with the negative control siRNA group. Importantly, the proliferation stimulating effect of GDNF on ISCs was significantly reduced in the NCAM siRNA transfected group compared with the negative control siRNA group (Figures 3H-L).

### ERK inhibitor PD98059 abolishes GDNF induced ISC proliferation

**Figure 4:**
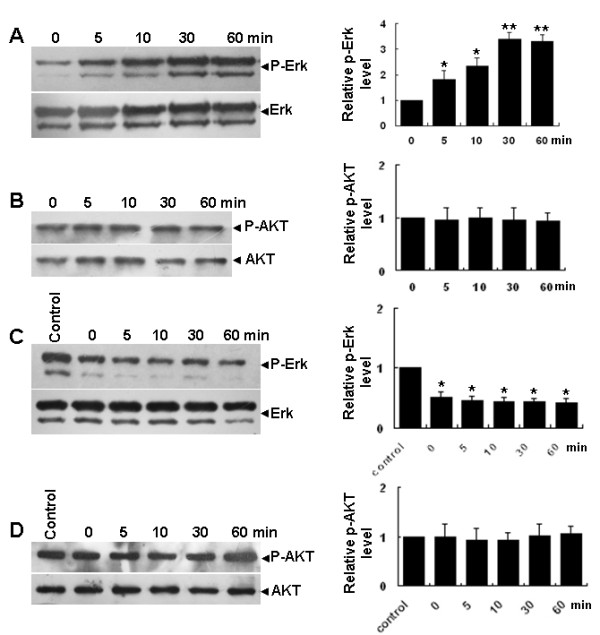
**Phosphorylation of ERK1/2 but not AKT in ISCs is upregulated by GDNF**. (A) Time-course of ERK1/2 phosphorylation levels in cultured ISCs stimulated with GDNF and the first bar (0) is cells without GDNF treatment. Total ERK1/2 was the internal control in the semi-quantitative densitometry analysis (right panel). (B) Time course of AKT phosphorylation levels in ISCs stimulated with GDNF, with total AKT as an internal control in the semi-quantitative densitometry analysis (right panel). (C) Time course of ERK1/2 phosphorylation level in PD98059 pre-treated (10 μM) ISCs stimulated with GDNF and the bar (control) is cells without GDNF or PD98059 treatment, with total ERK1/2 as the internal control in the semi-quantitative densitometry analysis (right panel). (D) Time course of AKT phosphorylation levels in PD98059 pre-treated (10 μM) ISCs stimulated with GDNF, with total AKT as the internal control in the semi-quantitative densitometry analysis (right panel). The data were presented as means ± SD from three independent experiments. Statistically significant differences (*p *< 0.05) among groups are indicated by an asterisk.

**Figure 5:**
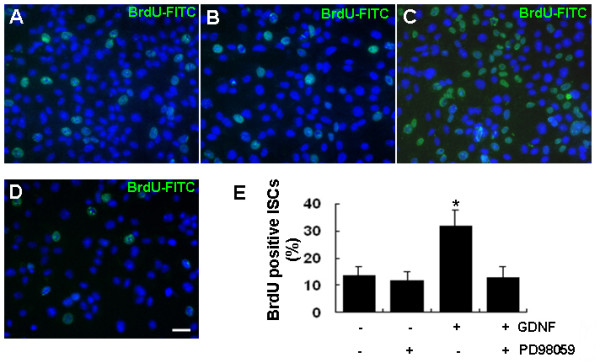
**Proliferation enhancement effects of GDNF on cultured ISCs is completely abolished by the ERK1/2 inhibitor PD98059**. (A-D) BrdU-positive cells in control (A), PD98059 treated (B), GDNF stimulated (C) and pre-treated with PD98059 plus GDNF stimulation groups (D). (E) Quantitative analysis results. The data were presented as means ± SD from three independent experiments. Statistically significant differences (*p *< 0.05) among groups are indicated by an asterisk. Scale bar indicates 10 μm.

It has been reported that GDNF plays an essential role in regulating the self-renewal of SSCs by activating the AKT and ERK1/2 signaling pathways [30,31]. To identify the signaling pathways activated by GDNF in ISCs, we first examined the phosphorylation levels of ERK1/2 and AKT in ISCs with and without GDNF treatment by Western blotting assays. The results showed that the phosphorylation level of ERK1/2 was significantly up-regulated 5 min post-GDNF stimulation and reached its highest levels after 30 min (Figure 4A). Notably, the increase of GDNF-induced ERK1/2 phosphorylation was completely blocked by pre-treatment of ISCs with the ERK1/2 inhibitor, PD98059 (10 μM) for 45 min (Figure 4C). The basal levels of Erk1/2 phosphorylation were also down-regulated by PD98059 treatment compared with the control group (Figure 4C). In contrast, GDNF stimulation (Figure 4B) or PD98059 pre-treatment and GDNF stimulation (Figure 4D) did not influence the phosphorylation level of AKT in ISCs. More importantly, PD98059 pre-treatment completely abolished GDNF stimulated proliferation of ISCs compared with the GDNF treatment group (Figures 5A-E).

## Discussion

Sertoli cells are the somatic cells in the testis that are essential for testis formation and spermatogenesis [1,32-34]. Proliferation of ISCs is important for male fertility because each Sertoli cell is able to support a limited number of germ cells [35,36]. Hormones such as FSH [37-39], estrogen [40] and thyroid hormones [41] as well as various paracrine growth factors including insulin-like growth factors I and II (IGF-I and IGF-II) [42], fibroblast growth factor (FGF) and somatomedin-C [43], activin [44], transforming growth factor-α (TGF-α) [45], and interleukin-1(IL-1) [46] are important in regulating the proliferation of ISCs. GDNF, a protein remotely related to the TGF-β super family members, and its receptor components GFRα1, RET and NCAM have been reported to be expressed in several types of testicular cells, implying a role of GDNF signaling in spermatogenesis. The role of GDNF signaling in spermatogonial proliferation and differentiation has been unequivocally demonstrated by *in vivo *studies using *Gdnf^+/- ^*mice and mice with specific over-expression of *Gdnf *in testis [5] and by *in vitro *studies in which GDNF was indentified as an essential factor for spermatogonial stem cell culture [4]. However, the role of GDNF in Sertoli cells is controversial. No Sertoli cell abnormality was reported in *Gdnf^+/- ^*mice and mice with *Gdnf *testis-specific over-expression while its stimulatory effect on the proliferation of Sertoli cells was observed in cultured neonatal rat seminiferous tubules and in Sertoli cells from fetal mouse testis [2,3,16]. In the present study, we used cultured ISCs from neonatal mice to show that GDNF stimulates the proliferation of ISCs derived from 4-5-day-old mice.

GFRα1 has been used as a specific surface marker for the identification and purification of mouse SSCs in some studies [29,47,48]. However, in the present study, we detected GFRα1 expression in cultured ISCs at both the mRNA and protein levels. As indicated by the results of He *et al*. [29,47,48], the protein level of GFRα1 in ISCs seemed to be much lower than in SSCs and was probably regarded as background signal in immunohistochemistry experiments by those authors. GDNF is known to signal through a multi-component receptor system consisting of GFRα1 and one of the two co-receptor subunits RET [7,8] or NCAM [14,49-51]. GFRa1 is the main ligand binding subunit and therefore is indispensible for the function of the receptor complex. Because the expression of RET was not detected in ISCs both at the mRNA and the protein levels, it is almost certain that the signal transduction is mediated by NCAM. Therefore, we focused on studying the role of NCAM in mediating GDNF signaling in ISCs although we could not fully exclude that additional or alternative receptors and pathways might also be involved in mediating the action of GDNF in ISCs. The essential role of NCAM in ISCs was supported by results from both NCAM antibody blocking and siRNA knockdown experiments. The observation that mRNA and protein of NCAM but not RET were expressed in cultured ISCs was consistent with one previous report [16].

It has been reported that GDNF signaling through the phosphorylation of ERK1/2 and AKT mediated the self-renewal division and proliferation of SSCs [28]. We observed in this study that GDNF stimulation also led to a rapid increase of ERK1/2 phosphorylation levels at Thr202/Tyr204 in cultured ISCs, but AKT phosphorylation levels did not change during this process. Significantly, the increase in ERK1/2 phosphorylation and ISC proliferation could be completely blocked by PD98059, with the phosphorylation level of AKT not changing during this process, suggesting that GDNF signaled through the ERK1/2 pathway in ISCs to execute its pro-proliferation function.

Proliferation of TM4 cells was not stimulated by GDNF although they possessed the same set of GDNF receptor subunits as ISCs. It is well known that TM4, an immortalized Sertoli cell line from mouse testis, has lost some characteristics of primary Sertoli cells. For example, FSH responsiveness was reduced in TM4 cells compared with primary Sertoli cell cultures [26]. We also noticed that CLU, a Sertoli cell marker, was not expressed by TM4 cells. Therefore, it is possible that downstream components of the GDNF signaling pathways have been changed in TM4 cells during its conversion from a primary culture to an immortalized cell line. Based on these characteristics of TM4, we used it as a negative control for studying ISC proliferation stimulated by GDNF in the present study. It will be instructive to elucidate the difference in GDNF signaling between ISCs and TM4 cells in future studies as the results would explain why TM4 cells and ISCs are different in response to GDNF treatment.

## Conclusions

The present study demonstrated that GDNF stimulates the proliferation of cultured mouse ISCs through its NCAM receptor subunit and the consequent activation of the ERK1/2 signaling pathway.

## Methods

### Reagents and Animals

Recombinant rat GDNF was obtained from R&D Systems (Minneapolis, MN, USA). A polyclonal rabbit anti-mouse NCAM and polyclonal goat anti-human vimentin were from Chemicon (Billerica, MA, USA). Polyclonal rabbit anti-mouse RET antibody (SC-167), rabbit anti-mouse GFRα-1 (H-70) and FITC-conjugated secondary antibodies were from Santa Cruz Biotechnology (Santa Cruz, CA, USA). A monoclonal mouse anti-BrdU antibody was from Hoffmann-La Roche (Basel, Switzerland). Polyclonal rabbit anti-ERK1/2, -phosphorylated ERK1/2, -AKT and -phosphorylated AKT antibodies were from Cell Signaling Technology (Beverly, MA, USA). The ERK inhibitor PD98059 was from Merck & Co. (Whitehouse Station, NJ, USA). Pre-designed mouse NCAM siRNA (AM16708, Ambion ID: 156439) and negative control siRNA (AM4611) was from Ambion (Norwalk, CT, USA).

ICR male mice (4-5-days-old) were used for ISC isolation and culture. Animals were treated in accordance with the NIH Guide for the Care and Use of Laboratory Animals. All the protocols were approved by the Animal Care and Use Committee of the Institute of Zoology of the Chinese Academy of Sciences.

### ISC cultures

Decapsulated testis tissue was treated with 1 mg/ml collagenase type IV and 1 mg/ml DNaseI (Sigma-Aldrich, St Louis, MO, USA) for 5 min with gentle agitation in Dulbecco's phosphate-buffered saline (DPBS) followed by three washes with DPBS to remove the testicular interstitial cells. Collected specimens were then treated with 0.25% trypsin-EDTA (Gibco, Carlsbad, CA, USA) and 1 mg/ml DNaseI for another 5 min in a 37°C water bath, and pipetted up and down with a 1 ml pipette to disperse the testicular cells. Subsequently, fetal bovine serum was added to a final concentration of 10% (v/v) to terminate the digestion. Cells were washed twice in DMEM/F12 medium containing 10% (v/v) FBS by suspending and centrifuging at 600 × g. The pellet was re-suspended in DMEM/F12 (Sigma-Aldrich) supplemented with 10% (v/v) FBS, 2 mM L-glutamine (Sigma-Aldrich), 100 units/ml penicillin and 100 units/ml streptomycin and plated on 0.2% (w/v) gelatin-coated tissue culture dishes at a density of 2 × 10^5 ^cells/cm^2^. Cells were cultured at 37°C/5% CO_2 _for 1 h. Floating cells were removed by gentle agitation and attached cells were cultured in FBS-free DMEM/F12 at 37°C/5% CO_2_. When cells were 90-95% confluent, cells were passaged. After being cultured in DMEM/F12 supplemented with 10% FBS for 1 h, floating cells were removed and the attached cells were cultured in FBS-free DMEM/F12. The purity of the cultured cells was assessed by immunocytochemistry and checking the ratio of vimentin-positive cells to DAPI-stained nuclei. Such cell cultures were designated as cultured ISCs of immature Sertoli cells. The TM4 cell line was cultured in DMEM/F12 medium containing 10% (v/v) FBS.

### ISC treatment and siRNA transfection

Cultured ISCs were pre-treated with 2 μg/ml NCAM antibody in culture medium for 1 h and the control group was pre-treated with 2 μg/ml non-specific rabbit IgG. For Erk1/2 inhibition, ISCs were pretreated with 10 μM PD98059 for 45 min. GDNF (20 ng/ml) was added to the medium in the presence of PD98059 and protein was collected at the indicated time points for Western blotting. For NCAM knockdown, siRNA specific to mouse NCAM and negative control siRNA were dissolved in nuclease-free water at 10 μM. ISCs and TM4 cells were transfected with NCAM siRNA and negative control siRNA using Lipofectamine™ 2000 (Invitrogen, Carlsbad, CA, USA) at a final concentration of 50 nM in accordance with the manufacturer's instruction. At 48 h post-transfection, cells were stimulated with GDNF for proliferation assays, or cells were collected to prepare proteins and RNAs for examination of NCAM mRNA and protein expression, respectively, in each group.

### Immunohistochemistry

Cultured cells on slides were stained according to a standard procedure. Briefly, cells were washed twice in PBS and fixed in 4% paraformaldehyde for 20 min at room temperature followed by another three washes in PBS. Cells were blocked with PBS containing 5% (w/v) bovine serum albumin (BSA) and 0.1% (v/v) Triton X-100 at room temperature for 60 min. The samples were then incubated overnight at 4°C with a primary antibody at a dilution recommended by the manufacturer. The slides were washed three times with PBS supplemented with 0.1% (v/v) Triton X-100 (PBST) for 15 min, incubated with diluted FITC-conjugated secondary antibody for 1 h at room temperature in the dark followed by another three washes with PBS. The nuclei of the cells were stained with DAPI (Sigma-Aldrich).

### Cell Proliferation Assay

Cells cultured on cover slips were stimulated with 20 ng/ml GDNF for 24 h and then pulsed with BrdU (BD Biosciences, Franklin Lakes, NJ, USA) at a final concentration of 10 μM in culture medium for 8 h. For the NCAM knockdown group, 48 h post-transfection, GDNF (20 ng/ml) was added directly to the culture medium and incubated for 24 h. BrdU was added to the culture system during the last 8 h at a final concentration of 10 μM. Cells were then fixed with ice-cold acetone-ethanol for 10 min on ice. Cell staining was performed according to the manufacturer's instructions (Roche). Briefly, fixed cells on slides were washed twice in PBS for 5 min each time. DNA denaturation was carried out by incubating the slides in 2 M HCl for 60 min at 37°C. The acid was neutralized by immersing the slides in 0.1 M borate buffer (pH 8.5) for 10 min with three changes of the buffer. Slides were washed with PBS three times for 10 min, placed in a humidified chamber, and cells covered with 150 μl of solution containing 3 μg/ml anti-bromodeoxyuridine diluted in PBS with 0.1% (w/v) BSA. After overnight incubation at 4°C, slides were washed with PBS three times for 15 min. Goat anti-mouse FITC-conjugated secondary antibody was added to the slides for 1 h at room temperature followed by three washes with PBS. Slides were incubated with 1 μg/ml DAPI, diluted in PBS, for 10 min at room temperature and were washed with PBS for 15 min at room temperature. Slides were mounted using glycerol and images were captured using an Olympus IX71 microscope.

### Reverse transcription-polymerase chain reaction (RT-PCR) and semi-quantitative analysis of gene expression

Total RNA was extracted from 5 × 10^5 ^Sertoli cells using Trizol (Invitrogen). First-strand cDNA was synthesized with M-MLV Reverse Transcriptase (Promega), and PCR was performed with recombinant Taq DNA Polymerase (Takara Bio Inc., Shiga, Japan) in accordance with the manufacturer's instruction. For semi-quantitative RT-PCR, equal amounts of RNA extracts were used to generate first-strand cDNAs. Primer pairs specific to mouse GDNF, GFRα1, RET, sulfated glycoprotein 2 [52] and NCAM cDNAs are listed in Table 1. The reference gene glyceraldehyde-3-phosphate dehydrogenase (G3PDH) was used as a normalization control. To perform the amplification, the reaction mixture was first denatured at 94°C for 3 min, followed by 35 cycles at 94°C for 1 min, 58°C for 40 s (65°C for Ret), then 72°C for 40 s. At the end, the reaction was incubated at 72°C for 7 min. For semi-quantitative RT-PCR, 30 cycles were used.

**Table 1:** Primer sequences and PCR products size

Gene	Forward primer	reverse primer	Product size
GDNF	5'-TCACTGACTTGGGTTTGGGCTAT-3'	5'-TCAGACGGCTGTTCTCACTCCTA-3'	477 bp
GFRα1	5'-ACTCCTGGATTTGCTGATGTCGG-3'	5'-CGCTGCGGCACTCATCCTT-3'	193 bp
Ret	5'-CTGCCGCTGCTAGGAGAAGCCCCAC-3'	5'-CTTCACACTGATGTTGGGACAAAGGAA-3'	555 bp
CLU	5'-GACAATGAGCTCCA(G/A)GAA(A/C)TG-3'	5'-CAGGCATCCTGTGGAGTT(G/A)TG-3'	806 bp
NCAM	5'-GCCGAGATGGTCATTCTGA-3'	5'-GATGTTGTCCAGGTGATGG-3'	571 bp
G3PDH	5'-ACCACAGTCCATGCCCATCAC-3'	5'-TCCACCACCCTGTTGCTGTA-3'	450 bp

### Western Blotting Analysis

Cells were harvested, washed in cold PBS, and homogenized at 4°C in lysis buffer (10 mM Hepes pH 7.9, 10 mM KCL, 1.5 mM MgCl_2_, 0.1 mM EGTA, 0.5 mM dithiothreitol, 10 mM glycerophosphate, 0.1 mM sodium vanadate and a pre-formed protease inhibitor mixture). Total cellular proteins were transferred to polyvinylidene difluoride membranes after SDS-PAGE. Membranes were blocked with PBS containing 5% (w/v) fat-free milk and 0.1% (v/v) Tween 20 for 1 h at room temperature and then hybridized with primary antibodies. After hybridization with secondary antibodies conjugated to horseradish peroxidase, the immunocomplexes were detected with Supersignal West Pico detection reagents (Pierce Protein Research Products, Rockford, IL, USA).

### Data analysis and statistics

Densitometry of semi-quantitative RT-PCR and Western blotting results were conducted using the Quantity One software with G3PDH and β-actin as internal controls, respectively. The values were presented as the mean ± standard deviation of three separate experiments. Statistically significant differences (*p *< 0.05 or *p *< 0.01) among groups were determined by one-way analysis of variance (ANOVA) and Tukey post-tests using SPSS (SPSS Inc, Chicago, IL, USA) statistical software.

## Authors' contributions

YGY performed the main experimental work, collection and assembly of data, data analysis and interpretation, and manuscript writing. CSH conceived and designed the experiments, conducted data analysis and interpretation, manuscript writing, and gave final approval of the manuscript. All authors read and approved the final manuscript.
